# Significant Clinical Factors Associated with Long-term Mortality in Critical Cancer Patients Requiring Prolonged Mechanical Ventilation

**DOI:** 10.1038/s41598-017-02418-4

**Published:** 2017-05-19

**Authors:** Li-Ta Keng, Kuei-Pin Chung, Shu-Yung Lin, Sheng-Kai Liang, Jui-Chen Cheng, I-Chun Chen, Yen-Fu Chen, Hou-Tai Chang, Chia-Lin Hsu, Jih-Shuin Jerng, Hao-Chien Wang, Ping-Hung Kuo, Huey-Dong Wu, Jin-Yuan Shih, Chong-Jen Yu

**Affiliations:** 1 Department of Internal Medicine, National Taiwan University Hospital Hsin-Chu Branch, Hsin-Chu, Taiwan; 2 Department of Laboratory Medicine, National Taiwan University Hospital, Taipei, Taiwan; 3 Department of Laboratory Medicine, National Taiwan University Cancer Center, Taipei, Taiwan; 4 Department of Internal Medicine, National Taiwan University Hospital, Taipei, Taiwan; 5 Department of Integrated Diagnostics and Therapeutics, National Taiwan University Hospital, Taipei, Taiwan; 6 Department of Oncology, National Taiwan University Hospital, Taipei, Taiwan; 7 Department of Oncology, National Taiwan University Cancer Center, Taipei, Taiwan; 8 Department of Internal Medicine, National Taiwan University Hospital Yun-Lin Branch, Yun-Lin, Taiwan; 9 Department of Internal Medicine and Department of Critical Care Medicine, Far Eastern Memorial Hospital, New Taipei, Taiwan

## Abstract

Studies about prognostic assessment in cancer patients requiring prolonged mechanical ventilation (PMV) for post-intensive care are scarce. We retrospectively enrolled 112 cancer patients requiring PMV support who were admitted to the respiratory care center (RCC), a specialized post-intensive care weaning facility, from November 2009 through September 2013. The weaning success rate was 44.6%, and mortality rates at hospital discharge and after 1 year were 43.8% and 76.9%, respectively. Multivariate logistic regression showed that weaning failure, in addition to underlying cancer status, was significantly associated with an increased 1-year mortality (odds ratio, 6.269; 95% confidence interval, 1.800–21.834; *P* = 0.004). Patients who had controlled non-hematologic cancers and successful weaning had the longest median survival, while those with other cancers who failed weaning had the worst. Patients with low maximal inspiratory pressure, anemia, and poor oxygenation at RCC admission had an increased risk of weaning failure. In conclusion, cancer status and weaning outcome were the most important determinants associated with long-term mortality in cancer patients requiring PMV. We suggest palliative care for those patients with clinical features associated with worse outcomes. It is unknown whether survival in this specific patient population could be improved by modifying the risk of weaning failure.

## Introduction

Acute respiratory failure is the leading cause of intensive care unit (ICU) admissions in critical cancer patients^1^. Although the hospital mortality in critical cancer patients has decreased to 30%, probably owing to advances in hemato-oncology and critical care medicine^2, 3^, it remains as high as 60–80% in those requiring mechanical ventilation^1, 4^. Many critically ill patients require prolonged mechanical ventilation (PMV) for post-intensive care^5^, and the burden of cancer patients requiring PMV support has rapidly increased in recent decades^6^. The survival of this population is extremely poor; the 1-year survival rate is 14.3%^7^.

Several prognostic factors, including organ failure, performance and cancer status, have been reported for mechanically ventilated cancer patients^8, 9^. Conversely, studies regarding prognostic assessment in cancer patients requiring PMV support are rare. Using data from the National Health Insurance Research Database in Taiwan, Shih *et al*. reported that patients with hepatic or pulmonary cancers or distant metastases show a worse survival, as compared to those with other cancer diagnoses or stages^7^. However, clinical and laboratory data were not included in the analyses performed by Shih *et al*. Potential clinical prognostic factors reported for general PMV patients, including disease severity, hemodialysis, and weaning status, may also be important for prognostic assessment in cancer patients requiring PMV, although they were neglected in Shih’s study based on a nationwide database^10–13^.

Accurate prognostic evaluation is mandatory for cancer patients requiring PMV, not only to avoid forgoing life-sustaining treatment for those with a chance of survival, but also to prevent futile medical care and poor end-of-life quality. Therefore, this study aimed to report the short- and long-term mortality rates of cancer patients requiring PMV in post-ICU settings, and to explore clinical factors significantly associated with weaning outcome and long-term mortality.

## Methods

### Settings and Population

This study was conducted in four medical ICUs in the National Taiwan University Hospital, a tertiary-care referral center in northern Taiwan. The study protocol was approved by the Institutional Review Board (NTUH REC: 201503008RINC), and the required informed consent was waived by the Institutional Review Board authority. All methods were performed in accordance with the relevant guidelines and regulations. The definition of PMV was ventilator support for >21 days, according to the regulations of the National Health Insurance in Taiwan and the definition from the National Association for Medical Direction of Respiratory Care Consensus Conference in 2005^14^. All patients requiring PMV support were transferred to the respiratory care center (RCC), a step-down and protocol-driven weaning facility, if the following criteria were not met: (1) age <20 years, (2) unresolved acute critical illness and ongoing multi-organ failure, (3) fraction of inspiratory oxygen (FiO_2_) ≥0.45 or positive end-expiratory pressure ≥8 cm H_2_O, and (4) unstable hemodynamic conditions, with requirement for high-dose vasopressors (dopamine ≥5 μg/kg/min or norepinephrine ≥5 μg/min). The eligibility of all patients requiring PMV before RCC transfer was screened from November 2009 to September 2013. Patients with a history of pathologically proven malignancies were enrolled. For patients who were admitted to the RCC more than once during the study period, only the first admission was included in the analysis.

### Data Collection

All clinical information and survival data were retrieved from patient medical records and the database of the Cancer Registry, Medical Information Management Office of the National Taiwan University Hospital. We defined newly-diagnosed cancers and cancers in progression as uncontrolled disease, while cancers in complete or partial remission or stable diseases after the last treatment, were defined as controlled disease. In patients with non-hematologic malignancies from two different origins, the disease status was defined as uncontrolled if either one was uncontrolled. The severity of illness, assessed by the Acute Physiology and Chronic Health Evaluation (APACHE) II^15^ and Sequential Organ Failure Assessment (SOFA) scores^16^, was determined at ICU admission and at the time of RCC transfer. The specific diagnoses at ICU admission were recorded and included severe sepsis or septic shock^17^, acute respiratory distress syndrome^18^, and acute kidney injury^19^. Nosocomial infections that occurred during the ICU stay were identified based on the Centers for Disease Control surveillance definition in 2014^20^. At RCC transfer, we recorded the presence of tracheostomy, the presence of active infection within 72 hours before transfer, and the results of various laboratory exams. The weaning parameters, including maximal inspiratory pressure (P_Imax_), maximal expiratory pressure (P_Emax_), rapid shallow breathing index, tidal volume, and minute ventilation (V_E_), were measured at RCC transfer and the results were interpreted using established cut-off values (P_Imax_, −20 cm H_2_O; P_Emax_, +30 cm H_2_O; rapid shallow breathing index, 105; tidal volume, 5 mL/kg; V_E_, 10 L/min)^21, 22^.

### Outcome Measurements

The outcome measurements in this study included the weaning status (success or failure) at RCC discharge, hospital mortality, and 1-year mortality. Weaning success was defined as independence from the ventilator (both invasive and non-invasive) for >5 days, according to the prospective payment system of ventilator dependents’ managed care by the National Health Insurance in Taiwan^7^. Patients were transferred to long-term care facilities (respiratory care wards) if they failed the protocol-driven weaning trials and were alive after a 6-week stay in the RCC. Weaning failure was considered in patients who failed mechanical ventilator disconnection at RCC discharge, in those who died during the RCC stay, and in those who returned to the ICU for aggravated critical illness. The weaning status and hospital mortality in non-cancer patients requiring PMV during screening were also recorded in comparison with those in cancer patients requiring PMV as in-house control.

### Statistical Analysis

Data are presented as the mean ± standard deviation for continuous variables and number (%) for categorical variables. Continuous variables were compared using Student’s *t*-test, while categorical variables were compared using Pearson’s χ^2^ or Fisher’s exact test, as appropriate. Kaplan–Meier curves were plotted for 1-year survival after RCC admission, and the differences between patient subgroups were compared using the log-rank test. Multivariate logistic regression analyses were performed to identify significant clinical characteristics associated with 1-year mortality or weaning outcome in the study population, as well as those associated with hospital mortality in all patients requiring PMV during screening. Significant variables in the univariate analyses were included in the models, and were backward selected with the entry and stay criteria set at 0.05 and 0.1, respectively. A two-sided *P* value < 0.05 was considered statistically significant. All analyses were performed using SPSS version 17.0 for Windows (IBM Corporation, Armonk, NY, USA).

## Results

### Study Population

During the study period, 5331 patients were admitted to the medical ICUs. Among 331 patients requiring PMV support who were subsequently transferred to the RCC, 112 (33.8%) had diagnoses of malignancies and constituted the study population. Eighty-nine (79.5%) patients had non-hematologic cancers, and 28 (25.0%) had hematologic cancers (Table 1). Five patients had diagnoses of both hematologic and non-hematologic cancers. The demographic features and clinical characteristics of the study population are shown in Tables 2 and 3.

**Table 1:** Hemato-oncologic diagnoses of the study population.

	Entire population		Survival outcome at 1 year (N = 108)				*P*
			Deceased		Survived		
N	112		83		25		
**Non-hematologic malignancy**	89	(79.5)	67	(80.7)	19	(76.0)	0.607
Cancer origin*							
Lung	24	(27.0)	21	(31.3)	3	(15.8)	0.182
Head and neck	20	(22.5)	12	(17.9)	6	(31.6)	0.213
Genitourinary tract	20	(22.5)	13	(19.4)	6	(31.6)	0.347
Gastrointestinal tract	14	(15.7)	13	(19.4)	1	(5.3)	0.178
Other	20	(22.5)	17	(25.4)	3	(15.8)	0.542
Disease status*							0.001
Controlled^†^	52	(58.4)	32	(47.8)	17	(89.5)	
Uncontrolled^†^	37	(41.6)	35	(52.2)	2	(10.5)	
**Hematologic malignancy**	28	(25.0)	20	(24.1)	7	(28.0)	0.693
Cancer histology^‡^							0.209
Leukemia	15	(53.6)	12	(60.0)	2	(28.6)	
Lymphoma or multiple myeloma	13	(46.4)	8	(40.0)	5	(71.4)	
Disease status^‡^							0.58
Controlled^†^	5	(17.9)	3	(15.0)	2	(28.6)	
Uncontrolled^†^	23	(82.1)	17	(85.0)	5	(71.4)	
**Double malignancies** ^§^	14	(12.5)	13	(15.7)	1	(4.0)	0.181

Data are presented as number (%). *Number (%) among patients with non-hematologic malignancy (n = 89). ^†^Disease conditions classified as cure, complete remission, partial remission, or stable disease were considered controlled, while those classified as progressive or newly-diagnosed diseases were considered uncontrolled. In patients with non-hematologic malignancies from two different origins, the disease status was defined as uncontrolled if either one was uncontrolled. ^‡^Number (%) among patients with hematologic malignancy (n = 28). ^§^Nine patients with solid cancers from two different origins, 4 patients with both solid cancer and hematologic malignancy, and 1 patient with hematologic malignancy and solid cancers from two different origins (triple malignancies).

**Table 2:** Clinical characteristics at baseline and during intensive care unit hospitalization.

	Entire population		Survival outcome at 1 year (N = 108)				*P*
			Deceased		Survived		
**N**	112		83		25		
**Age (years)**	69.0 ± 14.7		69.9 ± 14.5		66.7 ± 15.9		0.341
**Sex**							0.464
Male	65	(58.0)	50	(60.2)	13	(52.0)	
Female	47	(42.0)	33	(39.8)	12	(48.0)	
Cancer status*							0.005
Controlled non-hematologic cancer	52	(46.4)	32	(38.6)	17	(68.0)	
Uncontrolled non-hematologic cancer	37	(33.0)	35	(42.2)	2	(8.0)	
Without non-hematologic cancer	23	(20.5)	16	(19.3)	6	(24.0)	
**Co-morbidities**							
Congestive heart failure	13	(11.6)	9	(10.8)	4	(16.0)	0.493
Diabetes mellitus	35	(31.3)	29	(34.9)	6	(24.0)	0.306
Chronic lung disease	19	(17.0)	12	(14.5)	7	(28.0)	0.139
Cirrhosis	6	(5.4)	6	(7.2)	0	0	0.333
Neurologic disease	23	(20.5)	18	(21.7)	4	(16.0)	0.536
Chronic kidney disease	18	(16.1)	13	(15.7)	5	(20.0)	0.76
**ICU admission**							
APACHE II	27.0 ± 7.9		26.8 ± 7.5		28.0 ± 9.3		0.477
SOFA	7.5 ± 3.5		7.6 ± 3.5		7.0 ± 3.5		0.473
Severe sepsis/septic shock	57	(50.9)	45	(54.2)	9	(36.0)	0.11
Pneumonia	79	(83.2)	59	(83.1)	17	(81.0)	0.755
ARDS	22	(19.6)	19	(22.9)	3	(12.0)	0.236
Acute kidney injury^†^	30	(28.6)	22	(28.6)	8	(33.3)	0.656
**ICU stay**							
Infection^‡^	60	(53.6)	44	(54.2)	12	(48.0)	0.585
Severe sepsis/septic shock^‡^	21	(18.8)	16	(19.3)	4	(16.0)	>0.999
Length of stay (days)	27.7 ± 14.2		29.5 ± 15.5		23.0 ± 8.0		0.046

Data are presented as the mean ± standard deviation or number (%). APACHE II, Acute Physiology and Chronic Health Evaluation score; ARDS, acute respiratory distress syndrome; ICU, intensive care unit; SOFA, Sequential Organ Failure Assessment score. *Disease conditions classified as cure, complete remission, partial remission, or stable disease were considered under control, while those classified as progressive or newly-diagnosed diseases were considered uncontrolled. In patients with non-hematologic malignancies from two different origins, the disease status was defined as uncontrolled if either one was uncontrolled. ^†^In patients without end-stage renal disease before admission. ^‡^Hospital-acquired infection and hospital-acquired severe sepsis/septic shock.

**Table 3:** Clinical characteristics during respiratory care center hospitalization.

	Entire population		Survival outcome at 1 year (N = 108)					*P*
			Deceased			Survived		
**N**	112		83			25		
**RCC transfer**								
**Tracheostomy**	70	(62.5)	53		(63.9)	15	(60.0)	0.726
Active infection*	24	(21.4)	18		(21.7)	5	(20.0)	0.857
**APACHE II**	16.2 ± 5.7		16.9 ± 5.5			13.6 ± 4.9		0.007
**SOFA**	5.1 ± 2.8		5.7 ± 2.8			3.8 ± 2.0		0.001
**Laboratory examinations**								
Leukocytes (10^3^/μL)	10.5 ± 8.2		11.0 ± 9.2			9.3 ± 4.1		0.376
Platelets (10^3^/μL)	189.7 ± 139.0		178.2 ± 136.9			209.6 ± 131.5		0.313
Hemoglobin (g/dL)	9.4 ± 1.2		9.2 ± 1.0			9.9 ± 1.6		0.023
Creatinine (mg/dL)	1.4 ± 1.4		1.5 ± 1.5			1.1 ± 0.9		0.104
pH	7.4 ± 0.1		7.4 ± 0.1			7.4 ± 0.1		0.581
PaCO_2_ (mm Hg)	40.3 ± 9.4		39.2 ± 8.0			43.7 ± 12.0		0.086
PaO_2_/FiO_2_ (mm Hg)	290.7 ± 121.9		292.0 ± 120.2			277.8 ± 131.4		0.614
HCO_3_− (mmol/L)	27.2 ± 5.8		26.3 ± 5.5			29.6 ± 6.1		0.012
**Weaning parameter**								
P_Imax_ ≥ −20 cm H2O	20	(17.9)	15		(18.1)	4	(16.0)	> 0.999
P_Emax_ ≤ +30 cm H2O	53	(47.3)	38		(45.8)	13	(52.0)	0.585
RSBI ≥ 105	50	(44.6)	38		(45.8)	10	(40.0)	0.610
Tidal volume ≤ 5 mL/kg	49	(43.8)	35		(42.2)	11	(44.0)	0.871
Minute ventilation ≥ 10 L/min	31	(27.7)	28		(33.7)	3	(12.0)	0.035
**RCC stay**								
**Weaning success**	50	(44.6)	29		(34.9)	17	(68.0)	0.003
**Length of stay** (**days**)	20.0 ± 11.5		19.5 ± 11.8			22.0 ± 11.2		0.357

Data are presented as mean ± standard deviation or number (%). APACHE II, Acute Physiology and Chronic Health Evaluation score; FiO_2_, fraction of inspiratory oxygen; PaCO_2_, arterial partial pressure of carbon dioxide; PaO_2_, arterial partial pressure of oxygen; P_Emax_, maximal expiratory pressure; P_Imax_, maximal inspiratory pressure; RCC, respiratory care center; RSBI, rapid shallow breath index; SOFA, Sequential Organ Failure Assessment score. *Presence of active infection within 72 hours before RCC transfer.

### Significant Clinical Factors Associated with 1-year Mortality

**Figure 1:**
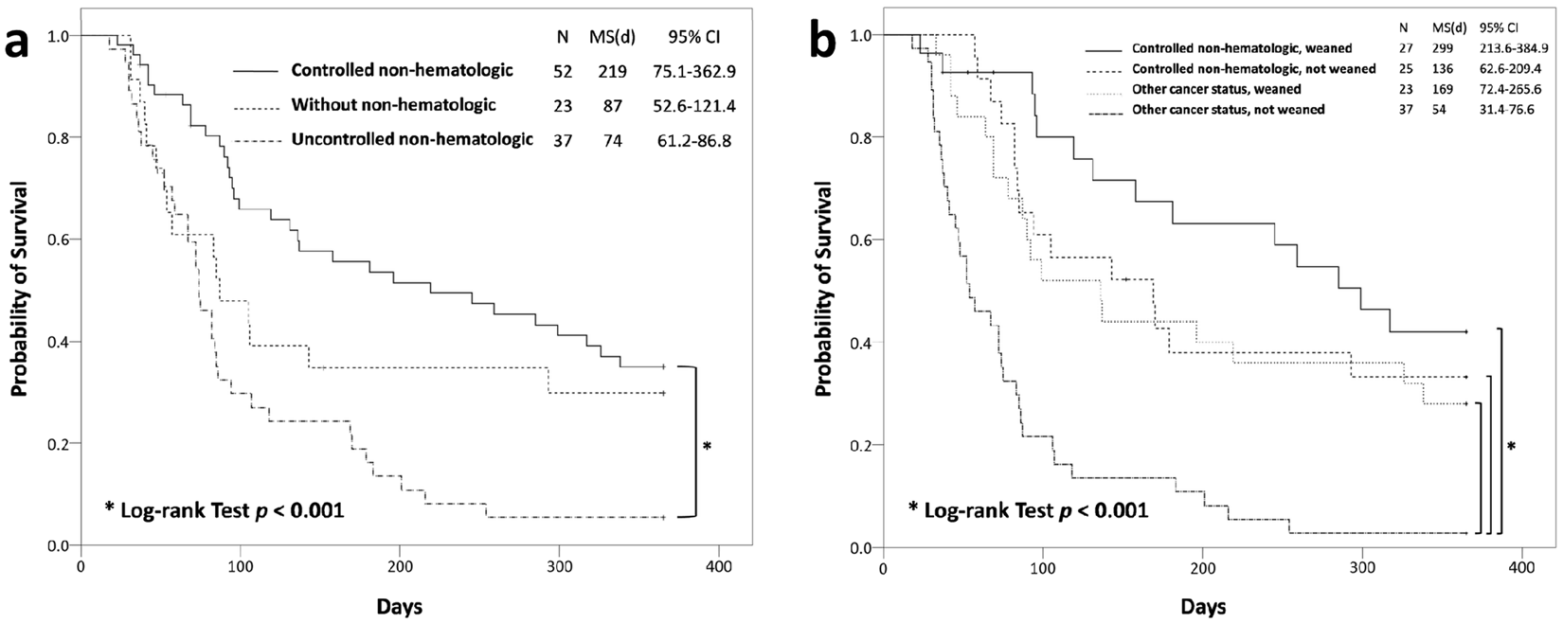
Kaplan-Meier curves for survival in cancer patients treated with prolonged mechanical ventilation, with stratification by cancer diagnosis and status (**a**), and weaning outcome and cancer status (**b**). CI, confidence interval; MS, median survival.

The mortality rate at hospital discharge was 43.8% (49/112). Compared to the screening 219 PMV patients without cancer during the study period, the PMV patients with cancer had a significantly lower survival to hospital discharge (56.3% vs. 77.6%, *P* < 0.001). Multivariate logistic regression analyses showed hematologic (odds ratio [OR], 3.148; 95% confidence interval [CI], 1.288–7.693; *P* = 0.012) and non-hematologic (OR, 2.756; 95% CI, 1.536–4.946; *P* = 0.001) malignancies were both independent clinical factors associated with an increased in-hospital mortality in all PMV patients (Supplementary Table S1). Survival information at 1 year was missing for 4 patients, and the 1-year mortality of the remaining 108 patients was 76.9% (83/108). Significant clinical factors associated with 1-year mortality were then evaluated for these 108 patients. Multivariate logistic regression analyses showed that an uncontrolled non-hematologic cancer status (OR, 11.779; 95% CI, 2.085–66.782; *P* = 0.005) and weaning failure (OR, 6.269; 95% CI, 1.800–21.834; *P* = 0.004) were the two most significant clinical factors associated with an increased 1-year mortality, followed by a lower HCO_3_^−^ level (Table 4). They remained significant when the 4 patients with missing data for survival status at 1 year were classified as dead (uncontrolled non-hematologic cancer status [OR, 7.079; 95% CI, 1.432–35.006; *P* = 0.016]; weaning failure [OR, 2.896; 95% CI, 1.005–8.347; *P* = 0.049]) or survived (uncontrolled non-hematologic cancer status [OR, 12.009; 95% CI, 2.136–67.533; *P* = 0.005]; weaning failure [OR, 6.039; 95% CI, 1.825–19.986; *P* = 0.003]). Kaplan-Meier survival curves were plotted for the different patient subgroups (Fig. 1). Patients with controlled non-hematologic cancers had a better survival compared with those with uncontrolled non-hematologic cancers or without non-hematologic cancers (Fig. 1a). We further divided the study population into subgroups according to weaning outcome and cancer status. We found that patients who had controlled non-hematologic cancers and successful weaning had the longest median survival time (299 days). Patients with hematologic or uncontrolled non-hematologic cancers who failed weaning had the worst median survival time (54 days) (Fig. 1b).

**Table 4:** Multivariate logistic regression models for significant clinical characteristics associated with 1-year mortality*.

Parameters	β	SE	Odds ratio (95% CI)		*P*
Hemato-oncologic status					
Non-hematologic cancer, under control			1		—
Non-hematologic cancer, uncontrolled	2.468	0.884	11.799	(2.085–66.782)	0.005
Without non-hematologic cancer	0.373	0.671	1.451	(0.390–5.405)	0.579
At the time of RCC transfer					
APACHE II score	0.105	0.063	1.111	(0.982–1.256)	0.094
HCO_3_^−^ (mmol/L)	−0.127	0.059	0.881	(0.784–0.989)	0.032
Minute ventilation (≥10 vs. <10 L/min)	1.367	0.743	3.925	(0.916–16.825)	0.066
Weaning outcome at RCC discharge (failure vs. success)	1.836	0.637	6.269	(1.800–21.834)	0.004

APACHE II, Acute Physiology and Chronic Health Evaluation score; CI, confidence interval; RCC, respiratory care center; SE, standard error. *Variables with statistical significance (*P* < 0.05) in the univariate analyses (Tables 1, 2, and 3) were included in the multivariate logistic regression models. Backward variable selection was performed, and the criteria of *P* values for entry and stay were set at 0.05 and 0.10, respectively.

### Significant Clinical Factors Associated with Weaning Failure

The weaning success rate at RCC discharge was 44.6% (50/112). Compared to the screening 219 PMV patients without cancer during the study period, the PMV patients with cancer had a similar weaning success rate (44.6% vs. 52.5%, *P* = 0.176). Since the weaning outcome was significantly related to 1-year mortality, we further explored the significant factors related to weaning. The clinical characteristics were compared between patients with successful weaning and those with weaning failure (Supplementary Tables S2–S4). Multivariate logistic regression analyses showed that low P_Imax_ (P_Imax_ ≥ −20 cm vs. <−20 cm H_2_O) (OR, 4.935; 95% CI, 1.409–17.278; *P* = 0.013) was the most significant clinical feature associated with weaning failure, followed by lower hemoglobin (OR, 0.619; 95% CI, 0.420–0.913; *P* = 0.016) and lower ratio of arterial oxygen partial pressure to fractional inspired oxygen (PaO_2_/FiO_2_) (OR, 0.996; 95% CI, 0.993–1.000; *P* = 0.040) (Table 5).

**Table 5:** Multivariate logistic regression models for significant clinical characteristics associated with weaning failure at respiratory care center discharge*.

Parameters	β	SE	Odds ratio (95% CI)		*P*
P_Imax_ (≥−20 vs. <−20 cmH_2_O)	1.596	0.639	4.935	(1.409–17.278)	0.013
Hemoglobin (g/dL)	−0.479	0.198	0.619	(0.420–0.913)	0.016
PaO_2_/FiO_2_ (mmHg)	−0.004	0.002	0.996	(0.993–1.000)	0.040

CI, confidence interval; FiO_2_, fraction of inspiratory oxygen; PaO_2_, arterial partial pressure of oxygen; P_Imax_, maximal inspiratory pressure; SE, standard error. *Variables with statistical significance (*P* < 0.05) in the univariate analyses (Supplementary Tables S2, S3 and S4) were included in the multivariate logistic regression models. Backward variable selection was performed, and the criteria of *P* values for entry and stay were set at 0.05 and 0.10, respectively.

## Discussion

A recent meta-analysis showed that in critically ill patients treated with PMV, the pooled mortality rates at hospital discharge and 1 year were 29% and 59%, respectively^23^. In this study, the hospital discharge mortality and 1-year mortality rates were 43.8% and 76.9%, respectively, in cancer patients requiring PMV support for post-intensive care. The 112 PMV patients with cancer had a significantly higher in-hospital mortality as compared to the screening 219 PMV patients without cancer during the study period. Furthermore, malignancies, whether hematologic or non-hematologic, were significant risk factors associated with in-hospital mortality in all patients receiving PMV support. These results are similar to those reported in Shih’s study based on a nationwide database^7^, and indicate that the prognosis in this specific clinical population is far worse than that for the general population requiring PMV. Although this and Shih’s studies both explore similar outcomes in cancer patients receiving PMV support, our results are from a hospital-based population, and disclose important prognostic clinical parameters, which couldn’t be evaluated using nationwide database. Therefore, our results offer clinicians important references for daily practice, and are complementary to the findings from the study based on the National Health Insurance database.

Although several clinical factors, including the APACHE II score and hemodialysis requirement, have been reported to be associated with survival in prolonged mechanically ventilated patients^10–13^, our study did not show that these clinical factors were significantly related to the survival of cancer patients requiring PMV. Our results indicate that the prognostic factors important for the general population requiring PMV cannot be directly applied to cancer patients requiring PMV. In cancer patients requiring mechanical ventilation, several studies have shown that cancer status is an independent risk factor for increased mortality^7–9, 24^. We further found that weaning outcome was significantly related to long-term survival in cancer patients requiring PMV, even in patients with an uncontrolled cancer status. Several studies have shown that the weaning protocol helps to improve weaning outcomes and survival in critically ill patients^25–28^. Weaning process standardization may also be beneficial in specific post-intensive weaning facilities, as in this study, since the result of weaning is not only an outcome measurement but also helps clinicians in prognostic assessment, particularly in cancer patients requiring PMV.

Besides cancer status and weaning outcome, our study showed that a higher HCO_3_^−^ level was independently associated with long-term survival in cancer patients requiring PMV. Studies exploring the relationship between HCO_3_^−^ levels and survival in critical care patients are rare. In a large retrospective study, the maximal serum HCO_3_^−^ level during ICU stay demonstrated a U-shaped association with in-hospital mortality, with the nadir at 29–30 mmol/L^29^. In our study, the mean HCO_3_^−^ level in 1-year survivors was within this range. Given that the upper limit of the 95% CI for HCO_3_^−^ level associated with 1-year mortality in the multivariate logistic regression model was very close to 1 (Table 4), further studies are required to clarify the relationship between the HCO_3_^−^ level and survival in critical cancer patients requiring PMV.

In cancer patients requiring PMV, our results showed that the impact of underlying malignancies on weaning outcomes were not as important as conditions associated with chronic critical illness and parameters related to respiratory function, including global inspiratory muscle strength (P_Imax_) and oxygenation (PaO_2_/FiO_2_ and hemoglobin)^22, 30^. Therefore, the malignancy diagnosis and status should not preclude the weaning trial, since successful weaning is associated with a better long-term survival. Several interventions aimed at improving weaning outcome in patients requiring mechanical ventilation have been proposed. Studies showed that a rehabilitation program in ventilator-bound patients helped to improve global and inspiratory muscle strength and weaning outcome^31, 32^. Although a subgroup analysis of the Transfusion Requirements in Critical Care trial showed no difference in the duration of mechanical ventilation between the restrictive and liberal transfusion strategies^33^, transfusion might facilitate weaning in the most debilitated patients with respiratory muscle weakness^34^. However, it is unknown whether these potential interventions can modify the risk of weaning failure in cancer patients requiring PMV support, and further studies are warranted.

Finally, early palliative care has been shown to improve the quality of life in advanced cancer patients^35^, and a transition from restorative to palliative treatment upon failure of the initial therapeutic trials has been advocated in the ICU^36^. However, the decision to make this transition depends on accurate prognostication. The study by Thiéry *et al*. demonstrated that the prognostic assessment at the time of ICU admission is often imprecise in critically ill cancer patients^37^. Our study also showed that conventional prognostic factors at the time of ICU admission, including the APACHE II and SOFA scores, as well as the admission diagnoses, were not significantly related to 1-year mortality in cancer patients requiring PMV. Therefore, the prognostic assessment may be more accurate if the clinical factors of post-intensive care, such as weaning outcome, are incorporated. Palliative care is highly suggested for patients with a poor cancer status and who failed weaning due to inevitably poor expected survival.

The present study had some limitations. First, the major limitation of this study was the retrospective design. Second, the study was conducted in a tertiary medical center and the major malignancy diagnoses were non-hematologic cancers. Therefore, the results may not be generalizable to other care settings, or to populations that are mainly constituted by patients with hematologic malignancies or bone marrow transplantation. Third, the number of patients with two types of cancer in this study was limited. It is unknown whether this population has a worse outcome than patients with a single cancer. Fourth, the withdrawal of mechanical ventilation was not legal in Taiwan until 2011. It is unknown whether the decision of ventilator withdrawal in the ICU will influence the clinical characteristics and outcomes in cancer patients treated with PMV, and follow-up studies are required.

In conclusion, the present study showed that cancer patients treated with PMV had high short-term and long-term mortality. Cancer status and weaning outcome were the most important determinants associated with long-term mortality. Neither cancer diagnoses nor status was significantly related to the weaning outcomes. Therefore, further weaning attempts in the specialized post-ICU weaning unit may not be precluded solely based on the underlying malignancy diagnoses or status, and palliative care may be considered in those with clinical features associated with worse outcomes. Future studies are required to evaluate whether the survival in this specific patient population can be improved by modifying the risk of weaning failure.

## Electronic supplementary material

Supplementary_Table_S1234
