# Supplementary material for: Significant Clinical Factors Associated with Long-term Mortality in Critical Cancer Patients Requiring Prolonged Mechanical Ventilation
Source: Sci Rep. 2017 May 19;7:2148. doi: 10.1038/s41598-017-02418-4 (PMC5438375; doi:10.1038/s41598-017-02418-4)
Supplement: Supplementary file 1 — Supplementary_Table_S1234 [file 41598_2017_2418_MOESM1_ESM.pdf]

Significant Clinical Factors Associated with Long-term Mortality in Critical Cancer Patients Requiring Prolonged Mechanical Ventilation

Li-Ta Keng[1], Kuei-Pin Chung[2,3], Shu-Yung Lin[4], Sheng-Kai Liang[1], Jui-Chen Cheng[5], I-Chun Chen[6,7], Yen-Fu Chen[8], Hou-Tai Chang[9], Chia-Lin Hsu[4]*, Jih-Shuin Jerng[4], Hao-Chien Wang[4], Ping-Hung Kuo[4], Huey-Dong Wu[4], Jin-Yuan Shih[4] & Chong-Jen Yu[4]

[1]Department of Internal Medicine, National Taiwan University Hospital Hsin-Chu Branch, Hsin-Chu, Taiwan.

Department of [2]Laboratory Medicine, [4]Internal Medicine, [5]Integrated Diagnostics and Therapeutics, and [6]Oncology, National Taiwan University Hospital, Taipei, Taiwan.

Department of [3]Laboratory Medicine and [7]Oncology, National Taiwan University Cancer Center, Taipei, Taiwan.

[8]Department of Internal Medicine, National Taiwan University Hospital Yun-Lin Branch, Yun-Lin, Taiwan.

[9]Department of Internal Medicine and Department of Critical Care Medicine, Far Eastern Memorial Hospital, New Taipei, Taiwan.

Supplementary Table S1. Multivariate logistic regression models for significant clinical characteristics associated with hospital mortality in all patients with prolonged mechanical ventilation*

**Parameters**	**β**	**SE**	**Odds ratio (95% CI)**	***P***
Hemato-oncologic diagnoses				
Non-cancer			1	–
Hematologic cancer	1.147	0.456	3.148 (1.288–7.693)	.012
Non-hematologic cancer	1.014	0.298	2.756 (1.536–4.946)	.001
At the time of ICU admission				
ARDS	0.097	0.365	2.711 (1.325–5.549)	.006
At the time of RCC transfer				
SOFA	0.139	0.056	1.149 (1.029–1.182)	.014
Leukocytes (10^3/μL)	0.057	0.021	1.058 (1.015–1.104)	.008
Hemoglobin (g/dL)	–0.346	0.134	0.708 (0.544–0.920)	.010
Bilirubin (mg/dL)	0.169	0.080	1.184 (1.013–1.384)	.034

ARDS, acute respiratory distress syndrome; CI, confidence interval; ICU, intensive care unit; RCC, respiratory care center; SE, standard error; SOFA, Sequential Organ Failure Assessment score.

*Variables with statistical significance ($P < .05$) in the univariate analyses were included in the multivariate logistic regression models. Backward variable selection was performed, and the criteria of *P* values for entry and stay were set at .05 and .10, respectively.

Supplementary Table S2. Hemato-oncologic diagnoses of the study population

	Entire population		RCC outcome				*P*
			Weaning success		Weaning failure		
**N**	112		50		62		
**Non-hematologic cancer**	89	(79.5)	40	(80.0)	49	(79.0)	.900
Cancer origin*							
Lung	24	(27.0)	7	(17.5)	17	(34.7)	.069
Head and neck	20	(22.5)	10	(25.0)	10	(20.4)	.606
Genitourinary tract	20	(22.5)	11	(27.5)	9	(18.4)	.305
Gastrointestinal tract	14	(15.7)	6	(15.0)	8	(16.3)	.864
Other	20	(22.5)	11	(27.5)	9	(18.4)	.305
Disease status*							.117
Controlled†	52	(58.4)	27	(67.5)	25	(51.0)	
Uncontrolled†	37	(41.6)	13	(32.5)	24	(49.0)	
**Hematologic malignancy**	28	(25.0)	14	(28.0)	14	(22.6)	.510
Cancer histology‡							.058
Leukemia	15	(53.6)	5	(35.7)	10	(71.4)	
Lymphoma or multiple myeloma	13	(46.4)	9	(64.3)	4	(28.6)	
Disease status‡							> .999
Controlled†	5	(17.9)	2	(14.3)	3	(21.4)	
Uncontrolled†	23	(82.1)	12	(85.7)	11	(78.6)	
**Double malignancies**§	14	(12.5)	8	(16.0)	6	(9.7)	.315

Data are presented as number (%). RCC, respiratory care center.

*Number (%) among patients with non-hematologic malignancy (n=89).

†Disease conditions classified as cure, complete remission, partial remission, or stable disease were considered controlled, while those classified as progressive or newly-diagnosed diseases were considered uncontrolled. In patients with non-hematologic malignancies from two different origins,

the disease status was defined as uncontrolled if either one was uncontrolled.

‡Number (%) among patients with hematologic malignancy (n=28).

§Nine patients with solid cancers from two different origins, 4 patients with both solid cancer and hematologic malignancy, and 1 patient with hematologic malignancy and solid cancers from two different origins (triple malignancies).

Supplementary Table S3. Clinical characteristics at baseline and during intensive care unit hospitalization

	Entire population		RCC outcome				*P*
			Weaning success		Weaning failure		
**N**	112		50		62		
**Age (years)**	69.0±14.7		69.1±12.6		69.0±16.3		.988
**Sex**							
Male	65	(58.0)	33	(66.0)	32	(51.6)	.125
Female	47	(42.0)	17	(34.0)	30	(48.4)	
**Cancer status***							.289
Controlled non-hematologic cancer	52	(46.4)	27	(54.0)	25	(40.3)	
Uncontrolled non-hematologic cancer	37	(33.0)	13	(26.0)	24	(38.7)	
Without non-hematologic cancer	23	(20.5)	10	(20.0)	13	(21.0)	
**Co-morbidities**							
Congestive heart failure	13	(11.6)	7	(14.0)	6	(9.7)	.478
Diabetes mellitus	35	(31.3)	11	(22.0)	24	(38.7)	.058
Chronic lung disease	19	(17.0)	7	(14.0)	12	(19.4)	.453
Cirrhosis	6	(5.4)	5	(10.0)	1	(1.6)	.087
Neurologic disease	23	(20.5)	11	(22.0)	12	(19.4)	.730
Chronic kidney disease	18	(16.1)	9	(18.0)	9	(14.5)	.618
**ICU admission**							
APACHE II	27.0±7.9		28.1±7.6		26.0±8.1		.168
SOFA	7.5±3.5		7.8±3.6		7.2±3.4		.317
Severe sepsis/septic shock	57	(50.9)	24	(48.0)	33	(53.2)	.582
Pneumonia	79	(83.2)	35	(83.3)	44	(83.0)	.968
ARDS	22	(19.6)	8	(16.0)	14	(22.6)	.384
Acute kidney injury[†]	30	(28.6)	17	(36.2)	13	(22.4)	.121

**ICU stay**							
Infection‡	60	(53.6)	30	(60.0)	30	(48.4)	.221
Severe sepsis/septic shock‡	21	(18.8)	10	(20.0)	11	(17.7)	.761
Length of stay (days)	27.7±14.2		27.1±15.2		28.2±13.3		.700

Data are presented as the mean±standard deviation or number (%). APACHE II, Acute Physiology and Chronic Health Evaluation score; ARDS, acute respiratory distress syndrome; ICU, intensive care unit; RCC, respiratory care center; SOFA, Sequential Organ Failure Assessment score.

*Disease conditions classified as cure, complete remission, partial remission, or stable disease were considered under control, while those classified as progressive or newly-diagnosed diseases were considered uncontrolled. In patients with non-hematologic malignancies from two different origins, the disease status was defined as uncontrolled if either one was uncontrolled.

†In patients without end-stage renal disease before admission.

‡Hospital-acquired infection and hospital-acquired severe sepsis/septic shock.

Supplementary Table S4. Clinical characteristics during respiratory care center hospitalization

	Entire population		RCC outcome				P
			Weaning success		Weaning failure		
**N**	112		50		62		
**RCC transfer**							
**Tracheostomy**	70	(62.5)	31	(62.0)	39	(62.9)	.922
**Active infection***	24	(21.4)	6	(12.0)	18	(29.0)	.029
**APACHE II**	16.2±5.7		15.8±5.0		16.5±6.2		.475
**SOFA**	5.1±2.8		4.5±2.5		5.7±2.9		.030
**Laboratory examinations**							
Leukocytes ($10^3/\mu L$)	10.5±8.2		9.4±5.0		11.3±10.1		.219
Platelets ($10^3/\mu L$)	189.7±139.0		214.2±145.9		170.0±131.0		.094
Hemoglobin (g/dL)	9.4±1.2		9.6±1.2		9.1±1.1		.032
Creatinine (mg/dL)	1.4±1.4		1.5±1.4		1.3±1.4		.475
pH	7.4±0.1		7.4±0.1		7.4±0.1		.671
$PaCO_2$ (mm Hg)	40.3±9.4		39.4±7.9		41.1±10.4		.318
PaO_2/FiO_2 (mm Hg)	290.7±121.9		317.4±127.1		269.1±114.0		.037
HCO_3- (mmol/L)	27.2±5.8		27.3±4.7		27.2±6.5		.929
**Weaning parameter**							
$P_{Imax} \geq -20$ cm H2O	20	(17.9)	4	(8.0)	16	(25.8)	.014
$P_{Emax} \leq +30$ cm H2O	53	(47.3)	20	(40.0)	33	(53.2)	.163
RSBI ≥ 105	50	(44.6)	18	(36.0)	32	(51.6)	.098
Tidal volume ≤ 5 mL/kg	49	(43.8)	19	(38.0)	30	(48.4)	.271
Minute ventilation ≥ 10 L/min	31	(27.7)	12	(24.0)	19	(30.6)	.435
**RCC stay**							
**Length of stay (days)**	20.0±11.5		19.0±10.0		20.7±12.5		.440

Data are presented as mean±standard deviation or number (%). APACHE II, Acute Physiology and Chronic Health Evaluation score; FiO_2, fraction of inspiratory oxygen; $PaCO_2$, arterial partial pressure of carbon dioxide; PaO_2, arterial partial pressure of oxygen; P_{Emax}, maximal expiratory pressure; P_{Imax}, maximal inspiratory pressure; RCC, respiratory care center; RSBI, rapid shallow breath index; SOFA, Sequential Organ Failure Assessment score.

*Presence of active infection within 72 hours before RCC transfer.
